# Supplementary material for: Spin current driven by ultrafast magnetization of FeRh
Source: Nat Commun. 2023 Jun 29;14:3619. doi: 10.1038/s41467-023-39103-2 (PMC10310832; doi:10.1038/s41467-023-39103-2)
Supplement: Supplementary file 1 — Supplementary Information [file 41467_2023_39103_MOESM1_ESM.pdf]

Supplementary Information

Spin current driven by ultrafast magnetization of FeRh

Kyuhwe Kang[1], Hiroki Omura[2], Daniel Yesudas[1], Oukjae Lee[3], Kyung-Jin Lee[4], Hyun-Woo Lee[5], Tomoyasu Taniyama[2], Gyung-Min Choi[1,6*]

[1]Department of Energy Science, Sungkyunkwan University, Suwon 16419, Korea

[2]Department of Physics, Nagoya University, Nagoya 464-8601, Japan

[3]Center for Spintronics, Korea Institute of Science and Technology, Seoul 02972, Korea

[4]Department of Physics, Korea Advanced Institute of Science and Technology, Daejeon 34141, Korea

[5]Department of Physics, Pohang University of Science and Technology, Pohang 37673, Korea

[6]Center for Integrated Nanostructure Physics, Institute for Basic Science, Suwon 16419, Korea

Section 1: FeRh thickness dependence on phase transition

The degree of the phase transition depends on the FeRh thickness. With FeRh thickness of 10 nm, the initial phase is not a complete AFM phase, but it is partially a FM phase with a small magnetization of 0.1×10^6 A m^{-1} at 300 K. Then, the quasi-static measurement shows that the magnetization change by the phase transition is rather a small value of 0.4×10^6 A m^{-1} (Fig. S1a). Increasing the FeRh thickness of 20 nm and 40 nm, the initial phase becomes a complete AFM phase with a negligible magnetization at 300 K. Then the magnetization change by the phase transition increases to 0.93×10^6 A m^{-1} (Fig. S1b,c). For the time-resolved measurement of ultrafast magnetization of FeRh and spin accumulation on Cu in the FeRh/Cu heterostructure, we used the FeRh thickness of 20 nm to have a complete AFM phase at 300 K.

Section 2: Magnetization Hysteresis curve of AFM and FM phases of FeRh

The magnetic hysteresis of AFM and FM phases of FeRh was investigated using a vibrating sample magnetometer. Magnetization of the FeRh 20 nm film was measured with the magnetic field along the in-plane direction (Fig. S2). At a base temperature of 300 K, FeRh becomes a complete AFM phase and shows no magnetization. At a base temperature of 430 K, FeRh becomes a complete FM phase and shows a saturation magnetization of 0.93×10^6 A m^{-1} and coercivity field of 15 mT.

Section 3: Optical setup for time-resolved detection

We used different optical setups depending on the experiments. Whereas the pump beam was fixed on the FeRh side of the sample, the probe beam was either on the FeRh side or Cu side depending on the experiment: probe on the FeRh side to monitor the phase transition of FeRh; probe on the Cu side to monitor the spin accumulation on Cu (Fig. S3). The probe measured the magnetization (ΔM) of the magnon bath (*d* band) of FeRh or spin accumulation (ΔS) of the conduction electron bath (*sp* band) of Cu *via* magneto-optical Kerr effect (MOKE) using a balanced detector. The sign of MOKE is determined by the magnetization direction of the FM phase of FeRh, which is set by the external magnetic field. We applied a magnetic field of 0.15 T along the out-of-plane direction using a ring magnet, whose shape allows passage of the pump and probe beams, to detect the z-components of ΔM and ΔS using polar MOKE. If a

longitudinal MOKE is preferred, one can apply the external field along the in-plane direction. Owing to the imperfection of the balanced detector, the raw signal has a small non-magnetic signal. To collect a pure magnetic signal, we simultaneously measured the Kerr rotation with +0.15 T and −0.15 T and took the difference. The probe also measured the lattice expansion of FeRh *via* the reflectivity change using a normal photodetector.

Section 4: Light absorption by FeRh

The light absorption by FeRh (a_{FeRh}) is shown as an attenuation of the Poynting vector (S), which is calculated using a transfer-matrix method with the refractive indexes of 1.73, 3+i5, and 0.3+i5 for MgO, FeRh, and Cu, respectively. When the light is incident on the FeRh side of the FeRh (20 nm)/Cu (120 nm) structure, through the MgO substrate, 55% is reflected at the MgO/FeRh interface, 44% is absorbed by the FeRh 20 nm, and 1 % is absorbed by the Cu 120 nm. The light absorption per thickness along the FeRh layer is obtained as dS/dz, where z is the position along the FeRh thickness (Fig. S4). The dS/dz is used for the distribution of the initial electronic heating in the thermal transport simulation and for the distribution of the spin generation in the spin transport simulation.

Section 5: Transient rising of temperature

The magnitude of the transient rising of temperature of a single FeRh layer by the pump pulse can be expressed as $\Delta T_{\mathrm{tran}} = \frac{a_{\mathrm{FeRh}} F_{\mathrm{in}}}{C_{\mathrm{FeRh}} d_{\mathrm{FeRh}}}$, where a_{FeRh} is the light absorption by FeRh, F_{in} is the incident pump fluence, C_{FeRh} is the heat capacity of FeRh, and d_{FeRh} is the thickness of FeRh. With a_{FeRh} of 0.4, F_{pump} of 7.1 J m^{-2}, C_{FeRh} of 3.2×10^{6} J m^{-3} K^{-1}, and d_{FeRh} of 20 nm, ΔT is 44 K. (The heat capacities of electron, magnon, and phonon baths of FeRh are taken from Ref. [1]. The light absorption is calculated in SI Section 4.). To estimate the dynamics of ΔT_{tran} in the FeRh (20 nm)/Cu (120 nm) heterostructure, we performed a heat transport simulation with an initial electronic heating by pump pulse (Fig. S5). Owing to a strong electron-phonon coupling of FeRh, ΔT of the phonon bath of FeRh reaches to 40 K at 1 ps. Therefore, the thermalization process alone cannot explain the time delay of 2.5 ps during the phase transition. For this thermalization simulation, we need to know the coupling parameters for the electron-phonon, electron-magnon, and magnon-phonon. Because these parameters are not known for FeRh, we

used typical values of conventional ferromagnet of Ni[2]: electron-phonon coupling of 10^{18} J m^{-3} K^{-1} and electron-magnon coupling of 0.5×10^{18} J m^{-3} K^{-1}. In addition, we also assume that magnon-electron coupling is much stronger than magnon-phonon coupling in FeRh. This assumption is often used for analysis of ultrafast demagnetization of FM and is supported by a slow demagnetization in insulating ferrimagnets without the conduction electron bath[3]. Despite the uncertainties on these coupling parameters, the thermalization simulation gives a rough estimation of the temperature dynamics. Material parameters for the thermalization simulation are summarized in Table S1.

Section 6: Steady state rising of temperature

In addition to the transient rise in temperature, a large pump fluence induces a significant rise in the steady-state temperature ($\Delta T_{\mathrm{steady}}$) of FeRh. The $\Delta T_{\mathrm{steady}}$ by the pump fluence is estimated as[4], $\Delta T_{\mathrm{steady}} = \frac{P_{\mathrm{in}} a_{\mathrm{FeRh}}}{2\sqrt{\pi} w_0 \Lambda}$, where P_{in} is the incident power of pump light, w_0 is the radius of pump light, and Λ is the thermal conductivity of MgO substrate. The P_{in} is related to the incident pump fluence (F_{in}), energy per area of a single pump pulse, as $F_{\mathrm{in}} = \frac{P_{\mathrm{in}}}{\pi w_0^2} \times \frac{2}{f_{\mathrm{EOM}}}$, where f_{EOM} is the modulation frequency of electro-optic modulator. Using F_{in} of 7.1 J m^{-2}, f_{EOM} of 1 MHz, w_0 of 3 um, a_{FeRh} of 0.4, and Λ of 30 W/mK, we estimate $\Delta T_{\mathrm{steady}}$ of ~10 K. With a much larger F_{in} of 28 J m^{-2}, $\Delta T_{\mathrm{steady}}$ is expected to be ~40 K. When $\Delta T_{\mathrm{steady}}$ becomes significant, the initial status of FeRh may consist of a small portion of ferromagnetic phase.

Supplementary References

1. Cooke, D. W., Hellman, F., Baldasseroni, C., Bordel, C., Moyerman, S., & Fullerton, E. E. Thermodynamic Measurements of Fe-Rh alloys. *Phys. Rev. Lett.* **109**, 255901 (2012).

2. Beaurepaire, E., Merle, J.-C., Daunois, A. & Bigot, J.-Y. Ultrafast spin dynamics in ferromagnetic nickel. *Phys. Rev. Lett.* **76**, 4250-4253 (1996).

3. Deb, M., Molho, P., Barbara, B., & Bigot, J.-Y. Controlling laser-induced magnetization reversal dynamics in a rare-earth iron garnet across the magnetization compensation point *Phys. Rev. B* **97**, 134419 (2018).

4. Cahill, D. G. Analysis of heat flow in layered structures for time-domain thermoreflectance,

Rev. Sci. Ins. **75**, 5119 (2004).

5. Choi, G. M., Moon, C. H., Min, B. C., Lee, K. J. & Cahill, D. G. Thermal spin-transfer torque driven by the spin-dependent Seebeck effect in metallic spin-valves. *Nat. Phys.* **11**, 576–581 (2015).
6. Tong, Z., Li, S., Ruan, X., & Bao, H. Comprehensive first-principles analysis of phonon thermal conductivity and electron-phonon coupling in different metals. *Phys. Rev. B* **100**, 144306 (2019).
7. Wang, W. & Cahill, D. G. Limits to thermal transport in nanoscale metal bilayers due to weak electron-phonon coupling in Au and Cu. *Phys. Rev. Lett.* 109, 175503 (2012).

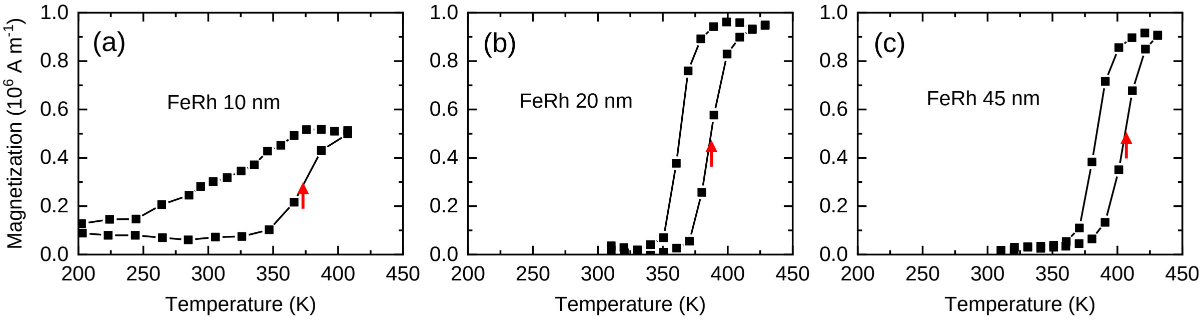

Figure S1. Magnetization (M) versus temperature of FeRh films. With the FeRh thickness of 10 nm (a), M of AFM phase at 300 K is ~0.1×10^6 A m^{-1}, and M of FM phase at 400 K is ~0.5×10^6 A m^{-1}. The non-zero M value of the AFM phase and rather small change of M between the AFM and FM phase suggest that the initial AFM ordering is not perfect. With the FeRh thickness of 20 nm (b) and 40 nm (c), M of AFM phase at 300 K is close to zero, and M of FM phase at 400 K is ~0.9×10^6 A m^{-1}. The negligible M value of the AFM phase and large change of M between the AFM and FM phases suggest that the initial AFM ordering is perfect. In addition, the critical temperature (red arrows) increases with the FeRh thickness: 375 K with 10 nm (a); 385 K with 20 nm (b); 405 K with 45 nm (c).

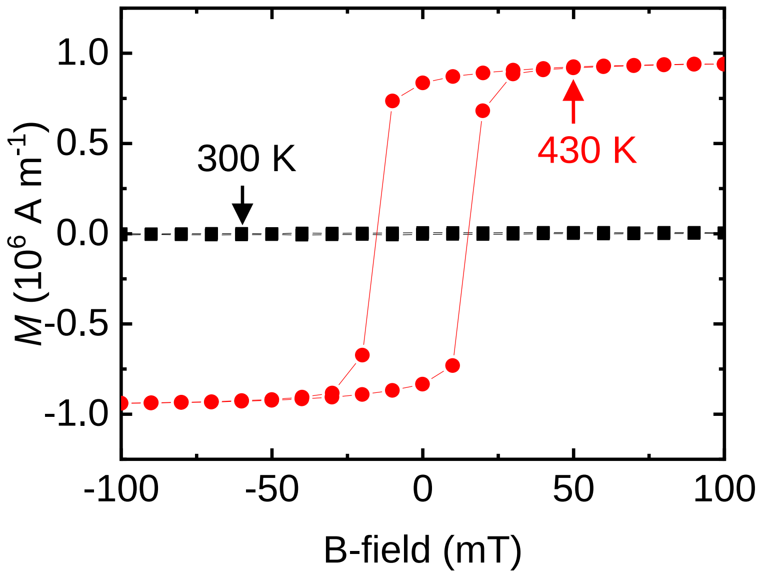

Figure S2. Magnetization (M) versus magnetic field (B-field) of FeRh films. M of FeRh 20 nm film was measured using a vibrating sample magnetometer with the B-field along the in-plane direction. At a base temperature of 300 K, FeRh becomes complete AFM phase and shows no magnetization. At a base temperature of 430 K, FeRh becomes complete FM phase and shows saturation magnetization of 0.93×10^6 A m^{-1} and coercivity field of ~15 mT.

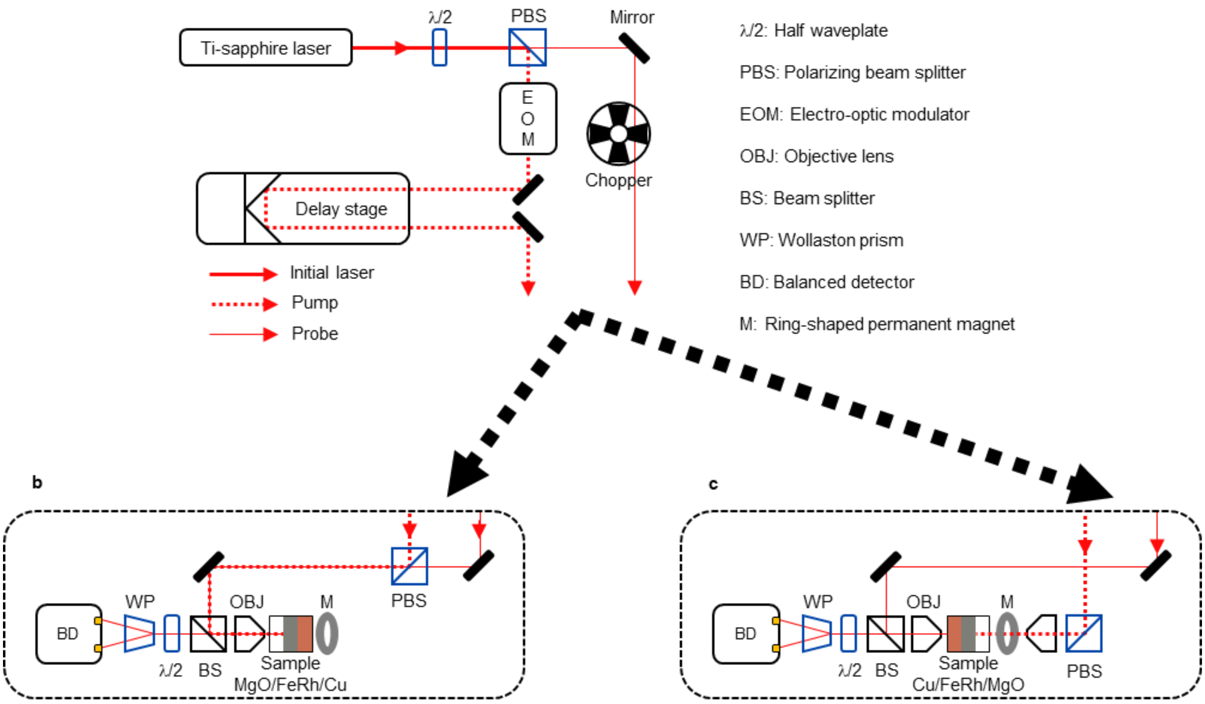

Figure S3. Schematics of optical setup. Ti-sapphire laser produces a pulsed laser with a wavelength of 785 nm. Polarizing beam splitter (PBS) split the laser beam into the pump (dotted line) and probe (solid line) beams. The pump and probe beams are modulated by electron-optic modulator (EOM) and chopper, respectively, at 1 MHz and 200 Hz. A delay stage controls a time delay between the pump and probe beams. Both pump and probe beams are focused on the sample surface using a 20X objective lens. The reflected probe beam passes a beam splitter (BS), halfwave plate (λ/2), and Wollaston prism (WP), then it is collected by a balanced detector (BD). To measure magnetization dynamics of FeRh, both pump and probe beams are on the FeRh side. To measure the spin accumulation on Cu, the probe is on the Cu side, and the pump is on the FeRh side. A ring magnet (M) is inserted between the sample and objective lens to align the magnetization of FeRh to the out-of-plane direction. Then, the magnetization dynamics and spin accumulation can be measured by polar MOKE.

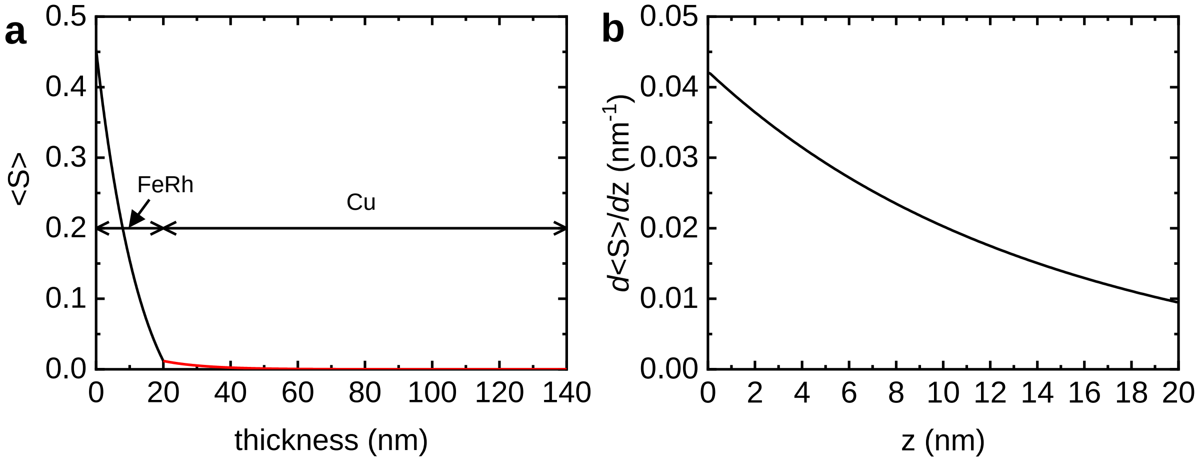

Figure S4. Light absorption in FeRh. (a) The attenuation of the light intensity in the MgO substrate/FeRh (20 nm)/Cu (120 nm) structure. We calculate the time-averaged Poynting vector, <S>, using a transfer-matrix method with the refractive indexes of 1.73, 3+*i*5, and 0.3+*i*5 for MgO, FeRh, and Cu, respectively. Most of the light absorption occurs in the FeRh layer (black line), and the Cu layer's contribution to the light absorption is negligible (red line). (b) The light absorption per thickness is calculated as the spatial derivative of <S> along the FeRh thickness (z). We assume that the initial electronic heating in the thermal transport simulation and the spin generation rate (g_s) in the spin diffusion simulation has the same distribution of (b) along the FeRh thickness.

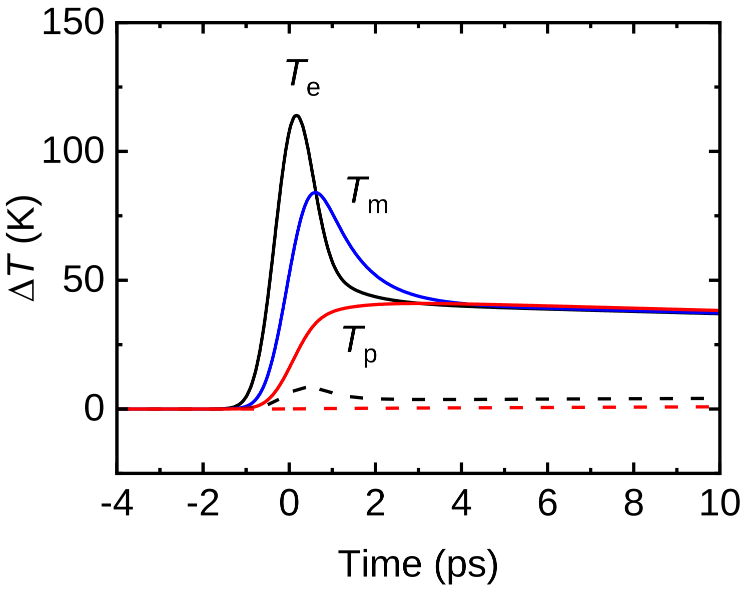

Figure S5. Dynamics of transient temperature rising in FeRh. The simulation of the temperature rising (ΔT) in the FeRh (20 nm)/Cu (120 nm) heterostructure with an incident pump fluence of 7.1 J m^{-2}. The back, red, and blue colors are for the electron (T_e), phonon (T_p), and magnon (T_m) temperatures, respectively. After the electronic heating by the pump pulse, there is rapid rising of T_p and T_m owing to the strong electron-phonon and electron-magnon couplings. The solid and dashed lines are for FeRh and Cu, respectively. A small-but-fast increase of T_e of Cu is due to the fast electronic transport from FeRh to Cu during the high T_e of FeRh. There is no rapid heating of Cu phonon because of the small electron-phonon coupling of Cu.

	AFM FeRh	Cu
C_{tot} (10^6 J m^{-3} K^{-1})	3.2	3.4
γ (J m^{-3} K^{-2})	215[a]	97[b]
C_m (10^6 J m^{-3} K^{-1})	0.3[a]	0
C_p (10^6 J m^{-3} K^{-1})	2.8[a]	3.4[b]
Λ_e (W m^{-1} K^{-1})	5.9[c]	300[b]
Λ_p (W m^{-1} K^{-1})	5[d]	5[d]
$g_{e\text{-}p}$ (10^{17} J m^{-3} K^{-2})	10[e]	0.75[f]
$g_{e\text{-}m}$ (10^{17} J m^{-3} K^{-2})	5[e]	

Table S1. Material properties for heat transport simulation. C_{tot} is the total heat capacity, γ is the electronic heat capacity coefficient, C_m is the magnon heat capacity, C_p is the phonon heat capacity, Λ_e is the electronic thermal conductivity, Λ_p is the phonon thermal conductivity, $g_{e\text{-}p}$ is the electron-phonon coupling, and $g_{e\text{-}m}$ is the magnon-phonon coupling.

a. from Reference[1].

b. from Reference[5].

c. obtained from Wiedemann-Frantz law with measured electrical conductivities.

d. typical value of metals from Reference[6].

e. a value of Ni from Reference[2].

f. from Reference[7]
